# Comparison of Clinical Performance of I-Gel™ with LMA—Proseal™ in Elective Surgeries

**Published:** 2009-06

**Authors:** Ishwar Singh, Monika Gupta, Mansi Tandon

**Affiliations:** 1 Chairperson, Department of Anesthesiology, Jaipur Golden Hospital, New Delhi, (INDIA); 2 Consultant, Department of Anesthesiology, Jaipur Golden Hospital, New Delhi, (INDIA); 3 P.G.Student, Department of Anesthesiology, Jaipur Golden Hospital, New Delhi, (INDIA)

**Keywords:** I-gel, LMA – ProSeal, Airway sealing pressure

## Abstract

**Summary:**

Sixty ASA grade I & II adult patients of either sex were randomly assigned into two groups. Group I (n=30) for I-gel and Group P (n=30) for LMA – ProSeal. We assessed the airway sealing pressure, ease of insertion, success rate of insertion, ease of gastric tube placement, airway trauma by post operative blood staining of the device, tongue, lip and dental trauma, hoarseness, regurgitation / aspiration and cost effectiveness. Although the airway sealing pressure was higher with Group P (29.6 cm H_2_O) than with Group I (25.27 cm H_2_0) (p < 0.05), but the airway sealing pressure of Group I was very well within the normal limit to prevent aspiration. The ease of insertion was more with Group I (29/30) than with Group P (25/30) (p < 0.05). The success rate of first attempt of insertion and ease of gastric tube placement was more with Group I (p > 0.05). Blood staining of the device & tongue, lip and dental trauma was more with Group P (p >0.05). There was no evidence of bronchospasm, laryngospasm, regurgitation, aspiration or hoarseness in either group.

To conclude I-gel is a novel supraglottic device with an acceptable airway sealing pressure (25.27 cm H_2_O). It is easier to insert, requires less attempts of insertion, has easier gastric tube placement and is less traumatic as compared to LMA-ProSeal.

## Introduction

**Fig 1:**
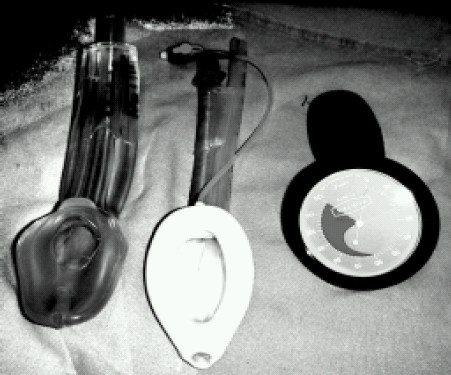
Showing I-gel, LMA-ProSeal and Pressure gauge (from left to right)

To overcome the limitations of currently available supraglottic airway devices like LMA-ProSeal (eg. high cost, demand for careful handling to prevent cuff damage and relative difficulty of insertion) a new and cheaper supraglottic airway device “I-gel”(Intetsurgical Ltd., Wokingham, Berkshire, UK) has been developed (Fig 1). I-gel is made up of medical grade thermoplastic elastomer, which is soft, gel like, transparent and designed to anatomically fit the perilaryngeal and hypopharyyngeaI structures without an inflatable cuff. It also has a port for gastric tube placement. I-gel is said to have easier insertion, minimal risk of tissue compression and stability alter insertion. It is a latex free supraglottic device. The buccal cavity stabilizer has a widened, elliptical, symmetrical and laterally flattened cross sectional shape, providing good vertical stability upon insertion which is an advantage over LMA with inflatable cuffs where mechanical inflation can cause movement of the device because the distal wedge shape of the mask is forced out of the upper oesophagus. The firmness of the tube section and its natural oropharyngeal curvature allows the device to be inserted by grasping the proximal end of I-gel and helps to glide the leading edge against the hard palate into the pharynx. It is not necessary to insert fingers into the mouth of the patient for full insertion. We compared I-gel and LMA – ProSeal in adults for the airway sealing pressure, ease of insertion, insertion attempts, ease of gastric tube placement, blood staining of the device, tongue, lip & dental trauma, bronchospasm/laryngospasm, regurgitation/aspiration and hoarseness.

## Methods

The present study was conducted in the Department of Anaesthesiology, Jaipur Golden Hospital, 2 institutional area, New Delhi after obtaining ethics committee clearance and written informed consent. Sixty ASA grade I and II adult patients of either sex scheduled for elective hernioplasty, laparoscopic cholecystectomy, tibial plating, humerus plating and skin grafting were selected. Patients with known difficult airway, cervical spine disease, mouth opening <2.5 cm, full stomach, hiatus hernia or gastroesophageal reflux disease & emergency surgeries were excluded from the study.

All the patients received injection midazolam 1mg, glycopyrrolate 0.2mg, ranitidine 50 mg and metoclopramide 10 mg intravenously 45 minutes before surgery. Anaesthesia was induced with propofol 2-2.5 mg.kg^−1^ and fentany 11–1.5μg.kg^−1^.Neuromuscular blockade was achieved with rocuronium 0.9 mg.kg^−1^. Both I-gel and LMA – ProSeal were lubricated with water soluble jelly. Once adequate depth was achieved each device was inserted by an experienced anaesthesiologist. Both the devices were fixed by taping the tube over the chin and lubricated gastric tube was placed into the stomach through the gastric channel. Maintenance was achieved by oxygen, nitrous oxide, isoflurane and intermittent doses of intravenous vecuronium. Intraoperative heart rate, noninvasive blood pressure, oxygen saturation and end tidal carbon dioxide were recorded before induction and at 1 and 5 minutes after insertion of device and then at every 5 minutes interval till the end of surgery.

An effective airway was judged by a square wave capnograph trace, normal thoraco - abdominal movement and absence of leak. If an effective airway could not be achieved the device was removed and three attempts were permitted before failure of insertion was recorded. If three attempts were unsuccessful either an alternative device was inserted or the trachea was intubated. The number of insertion attempts was recorded.

The ease of insertion of device was also recorded. Ease was defined as no resistance to insertion in the pharynx in a single maneuver. In a difficult insertion there was resistance to insertion or more than one maneuver was required for the correct placement of the device.

The ease of placement of gastric tube was also recorded and its correct placement was confirmed by injection of air and epigastric auscultation or aspiration of gastric contents. Failure of gastric tube placement was also recorded and it was defined as failure to advance the gastric tube into the stomach with in two attempts.

The airway sealing pressure was determined by closing the expiratory valve of the circle system at a fixed gas flow of 3 L/minute and recording the airway pressure (Cuff inflator/pressure gauge from Portex Germany) at which equilibrium was achieved. At this time gas leakage was determined at the mouth by the audible leak or by detection of an audible noise using a stethoscope placed just lateral to thyroid cartilage. At the end of surgical procedure anaesthesia was discontinued, patient was reversed with standard dose of neostiamine and glycopyrrolate and the device was removed. Blood staining of the device and tongue, lip and dental trauma were recorded. Regurgitation of gastric contents was also assessed. Pharynaolaryngeal morbidity was assessed as hoarseness of voice in the post-anaesthesia care unit and 24 hours subsequently.

The sample size was based on a crossover pilot study of 10 patients and was selected to detect a projected difference of 30% between the groups for airway sealing pressure for type I error of 0.05 and a power of 0.8. Statistical analysis for airway sealing pressure was done by Fisher's t–test. For the two variables, ease of insertion of gastric tube & bronchospasm/laryngospasm dichotomous nominal scale data correlation was applied. For the remaining characteristics Chi square test with Yate's correction was applied. Significance was taken as p < 0.05.

## Results

There was no difference between the two groups with respect to demographic and surgical details. (Table 1) In all patients the supraglottic device, I-gel or LMA – ProSeal, was inserted within three attempts. The average airway sealing pressure with I-gel was 25.27 cm H_2_O and that with LMA – ProSeal was 29.6 cm H_2_O which was statistically significant (p<0.05) (Table 2). The ease of insertion was more with I-gel (29/30) than with LMA – ProSeal (23/30) which was statistically significant (p<0.05) (Table 2). The success rate at first attempt of insertion were 30/30 (100%) for I-gel & 28/30 (93.3%) for LMA – ProSeal which was statistically not significant. (Table 2). The ease of insertion of gastric tube was more with I-gel (30/30) than with LMA – ProSeal (26/30) (Table 3). Tongue, lip & dental trauma was more with LMA – ProSeal (5/30) than with I-gel (1/30) and blood staining of the device was more with LMA– ProSeal (6/30) than with I-gel (1/30) but the results were not statistically significant (Table 3). There was no incidence of bronchospasm / laryngospasm, aspiration / regurgitation and hoarseness in both the groups. (Table 3)

**Table 1:** Demographic data(Mean±SD or n)

Particulars	I-gel	LMA – Pro Seal
Age (yrs)	38.31 ± 12.24	39.86 ± 13.08
Weight (kg)	60.24 ± 10.89	61.27 ±11.85
Gender		
Male (n1)	15	13
Female (n2)	15	17
N = n1 + n2	30	30
Type of surgery		
Hernioplasty	10	11
Lap.cholecystectomy	6	7
Tibial plating	8	7
Humerus plating	3	3
Skin grafting	3	2

**Table 2:** Comparison of airway sealing pressure, ease of insertion and insertion attempts

Parameters	I-gel	LMA - ProSeal	p–value
Airway sealing pressure (cm H_2_O)	25.27(6.44)	29.6(5.62)	<0.05
Average(SD)			
Ease of insertion(n)			
Easy	29	23	<0.05
Difficult	1	7	
Insertion attempts(n)			
1	30	28	>0.05
2	0	2	
3	0	0	
Failed	0	0	

**Table 3:** Comparison of other parameters

Parameters	I-gel	LMA - ProSeal	p–value
Ease of gastric tube insertion			
Easy	30	26	>0.05
Difficult	0	4	
Failed	0	0	
Blood staining of device			
Yes	1	6	>0.05
No	29	24	
Tongue–lip–dental trauma			
Yes	1	5	>0.05
No	29	25	
Bronchospasm/laryngospasm	0	0	
Hoarseness	0	0	
Regurgitation/aspiration	0	0	

## Discussion

We found, I-gel to be as effective as LMA–ProSeal in providing patent airway during controlled ventilation of lungs. The airway sealing pressure was higher with LMA – ProSeal (29.6 cm H_2_O) than with I-gel (25.27 cm H_2_O) a statistically significant finding but the airway sealing pressure of I-gel was also within normal limit and effective in preventing aspiration. In our study the sealing pressure was measured by closing the expiratory valve of the circle system at a fixed fresh gas flow of 3 L /minute until airway pressure reached a steady value^2^,^3^. Keller C, et al^4^ and Lopez -Gil et al^5^ compared four kinds of measurements of the airway sealing, pressure, which involved detection of audible noise by listening over the mouth, detection of exhaled carbon dioxide by placing a gas sampling line for the capnograph inside the mouth, detection of a steady value airway pressure while occluding the expiratory, valve of the circle system and detection of an audible noise using a stethoscope placed just lateral to the thyroid cartilage. They concluded that all four tests were excellent.

The ease of insertion was more with I-gel (29/30) than with LMA – ProSeal (23/30) which was a statistically significant difference. Levitan & Kinkle^1^ presumed that on insertion of LMA with inflatable mask the deflated leading edge of the mask can catch the edge of the epiglottis & cause it to downfold or impede proper placement beneath the tongue. Brimacombe and colleagues^6^,^7^ presumed that the difficulties in inserting LMA – ProSeal were caused by larger cuff impeding digital intra – oral positioning and propulsion into the pharynx, the lack of a backplate making cuff more likely to fold over at the back of mouth and the need for more precise tip positioning to prevent air leaks up the drainage tube.

Incidence of blood staining of the device was more with LMA – ProSeal (6/30) than with I-gel (1 /30) & tongue, lip & dental trauma was more with LMA – ProSeal (5/30) than with I-gel (1/30) which was otherwise statistically not significant. Levitan & Kinkle^1^ presumed that inflatable masks have the potential to cause tissue distortion, venous compression & nerve injury. The ease of gastric tube placement was more with I-gel (30/30) than with LMA – ProSeal (26/30), though the difference was not statistically significant. Also there was no statistical difference between the insertion attempts of the devices. Both I-gel and LMA – ProSeal had no incidence of bronchospasm/laryngospasm, aspiration/regurgitation and hoarseness.

To conclude the I-gel is a cheap and effective device which is easier to insert (statistically significant as compared to LMA – ProSeal). It has other potential advantages like effective airway sealing pressure which was within the normal limit, easier gastric tube placement, less blood staining of the device and less tongue, lip and dental trauma.

In our opinion the I-gel is a useful supraglottic device.
